# House Dust Mite Allergen Regulates Constitutive Apoptosis of Normal and Asthmatic Neutrophils via Toll-Like Receptor 4

**DOI:** 10.1371/journal.pone.0125983

**Published:** 2015-05-14

**Authors:** Do Hyung Kim, Eugene Choi, Ji-Sook Lee, Na Rae Lee, Seung Yeop Baek, Ayoung Gu, Da Hye Kim, In Sik Kim

**Affiliations:** 1 Department of Biomedical Laboratory Science, School of Medicine, Eulji University, Daejeon 301–768, Republic of Korea; 2 Department of Respiratory Internal Medicine, College of Medicine, Konyang University, Daejeon, 302–718, Republic of Korea; 3 Department of Clinical Laboratory Science, Wonkwang Health Science University, Iksan, 570–750, Republic of Korea; 4 Department of Senior Healthcare, BK21 plus program, Graduate School, Eulji University, Daejeon 301–746, Republic of Korea; National Institute of Environmental Health Sciences, UNITED STATES

## Abstract

House dust mites (HDMs) induce allergic diseases such as asthma. Neutrophil apoptosis is an important process of innate immunity, and its dysregulation is associated with asthma. In this study, we examined the effects of HDM on constitutive apoptosis of normal and asthmatic neutrophils. Extract of *Dermatophagoides pteronissinus* (DP) inhibited neutrophil apoptosis, but *Dermatophagoides farinae* extract had no effect. Anti-apoptotic signaling mediated by DP involves in TLR4, Lyn, PI3K, Akt, ERK, and NF-κB in normal neutrophils. DP delayed cleavage of procaspase 9 and procaspase 3 and the decrease in Mcl-1 expression. Supernatant collected from DP-treated normal neutrophils inhibited the constitutive apoptosis of normal neutrophils, and S100A8 and S100A9 were identified as anti-apoptotic proteins in the supernatant. S100A8 and S100A9 transduced the anti-apoptotic signal via TLR4, Lyn, PI3K, Akt, ERK, and NF-κB. DP also suppressed asthmatic neutrophil apoptosis and induced secretion of S100A8 and S100A9, which delayed the constitutive apoptosis. The anti-apoptotic effects of DP, S100A8 and S100A9 in asthmatic neutrophils are associated with TLR4, Lyn, PI3K, Akt, ERK, and NF-κB. The concentrations of S100A8 and S100A9 were significantly elevated in asthmatic bronchoalveolar lavage fluid (BALF) when compared to normal BALF (*p*<0.01), but not in serum. S100A8 concentration in BALF was positively correlated with the number of BALF neutrophils and negatively correlated with FEV1(%). These findings improve our understanding of the role of HDM in regulation of neutrophil apoptosis in normal individuals and asthmatics and will enable elucidation of asthma pathogenesis.

## Introduction

Asthma is an allergic disease characterized by airway obstruction, allergen-specific IgE and bronchial inflammation. House dust mites (HDMs), which primarily consist of *Dermatophagoides pteronissinus* (DP) and *Dermatophagoides farinae* (DF), are heavily involved in asthma pathogenesis [1, 2]. HDM allergens trigger allergic inflammation via Toll-like receptor (TLR), C type lectin receptor, NOD-like receptor and proteinase-activated receptor signaling pathways, and TLR4 in the TLR signaling is important in HDM-induced asthma [3, 4]. Asthma is composed of neutrophilic and eosinophilic subtypes. Neutrophilic asthma is characterized by a persistence of airway neutrophilia and is responsible for approximately half of mild-to-moderate asthmatic subjects. Neutrophil recruitment to the airway is associated with airway hyper-responsiveness, and neutrophilic asthmatics show a poor response to inhaled corticosteroids [5, 6]. Accordingly, neutrophils are considered important pathogenic agents and a worthy target in asthma treatment [5, 7, 8].

During inflammation or infection, neutrophils move toward the inflamed site and function as immune regulators by interacting with causative factors [9, 10]. The plasticity of the neutrophil life span is accomplished by inhibiting apoptosis by extracellular ligands such as GM-CSF, tumor necrosis factor-α (TNF-α) and CCL2 secreted from immune and structural cells [11, 12, 13]. However, the inhibition of constitutive neutrophil apoptosis induces or aggravates pathogenic lesions via persistent accumulation of neutrophils in inflammatory diseases such as asthma [14, 15]. Delayed neutrophil apoptosis is associated with the phosphatidylinositol 3-kinase (PI3K)/Akt pathway and cAMP/protein kinase A pathway [10].

In this study, we studied the role of HDM in constitutive neutrophil apoptosis in normal and asthmatic subjects, as well as common anti-apoptotic mechanisms of the HDM-directed pathway and the pathway mediated by anti-apoptotic proteins secreted due to HDM.

## Materials and Methods

### Reagents

RPMI 1640 and fetal bovine serum (FBS) were purchased from Life Technologies Inc. (Gaithersburg, MD). DP and DF extracts were obtained from Cosmo Bio (Tokyo, Japan) and the Korea National Arthropods of Medical Importance Resource Bank (Yonsei University, Seoul, Korea). Endotoxin level was measured by Limulus amebocyte lysate QCL-1000 test (Lonza, MD) and the level is low (< 0.5 ng/ml). CLI-095, an inhibitor of Toll-like receptor (TLR) 4 (TLR4i), was purchased from Invivogen (San Diego, CA, USA). Src family protein inhibitor (PP2), PI3K inhibitor (Ly294002), Akt inhibitor (AKTi), MEK inhibitor (PD98059), and NF-κB inhibitor (BAY-11-7085) were acquired from Calbiochem (San Diego, CA, USA). Antibodies against phospho-Lyn, Lyn, phospho-ERK1/2 and Mcl-1 were purchased from Cell Signaling Technology (Beverly, MA, USA). Antibodies against phospho-Akt, Akt, ERK2, procaspase 3, and procaspase 9 were obtained from Santa Cruz Biotechnology (Santa Cruz, CA, USA). Anti-S100A8 and S100A9 antibodies were obtained from Abnova (Taipei Taiwan). IFN-γ, IL-4, IL-9, IL-10, IL-17, and TGF-β were obtained from R&D Systems (Minneapolis, MN, USA).

### Normal subjects and asthmatic patients

A total of 184 asthmatic patients with mild to severe symptoms were recruited from Konyang University Hospital (Table 1). Additionally, 93 normal subjects were recruited as controls. The normal subjects had normal lung function, no history of asthma, and did not require medication. This study was approved by the Institutional Review Board of Eulji University for normal volunteers and by the Institutional Review Board of Konyang University for asthma patients. All participants in this study gave their written informed consent.

**Table 1:** Patient characteristics.

	Normal	Asthma
**Number of subjects (female/male)**	93 (49/44)	184 (106/78)
**Age (years)**	25 ± 4.5 (18~54)	52 ± 18.5 (15~90)
**FEV1** ^**§**^ **(% predicted)**	101.6±11.9 (87.1~121.3)	81.3±23.3* (33.5~133.0)
**FVC** ^∥^ **(% predicted)**	95.2±11.5 (77.9~112.9)	91.1±17.7 (47.9~139.5)
**FEV1/FVC (%)**	91.6±4.9 (84.8~97.9)	70.6±14.6** (32.0~98.0)

FEV1^§^: forced expiratory volume in one second

FVC^∥^: forced vital capacity

Data are expressed as the means SD (the lowest value ~ the highest value)

*p < 0.05 and **p < 0.01 indicate statistically significant differences between the normal and asthma groups.

### Neutrophil isolation and cell culture

Human neutrophils were isolated from the heparinized peripheral blood of healthy persons and asthmatics using Ficoll-Hypaque gradient centrifugation and a CD16 microbeads magnetic cell sorting kit (Miltenyi Biotec, Bergisch Gladbach, Germany). The cells were washed after hypotonic lysis to remove erythrocytes and then resuspended at 3 x 10^6^/ml in RPMI 1640 medium with 1% penicillin-streptomycin and 10% FBS. Counting the cells on cytospin revealed that this method routinely yielded greater than 97% neutrophil purity.

### Detection of apoptosis

An annexin V-fluorescein isothiocyanate (FITC) apoptosis detection kit (BD Biosciences, San Diego, CA, USA) was used to detect neutrophil apoptosis. Isolated neutrophils were incubated with an FITC-labeled annexin V and propidium iodide (PI) for 15 min at room temperature. Apoptotic neutrophils were analyzed using a FACSCalibur with CellQuest software (BD bioscience) and were determined as the percentage of cells showing annexin V+/PI- and annexin V+/PI+. For the morphological estimation of neutrophil apoptosis, neutrophils were cytocentrifuged and stained with Wright staining solution.

### Western blotting

After being treated with DP, S100A8, or S100A9, neutrophils were harvested and lysed in a cytosolic extraction buffer. The homogenate was then centrifuged at 10,000 g for 1 min at 4°C, after which the supernatant was collected as a cytosolic fraction. The pellet was then resuspended in 50 μl of nuclear extraction buffer and centrifuged at 12,000 g for 15 min at 4°C, after which the supernatant was collected as a nuclear fraction. The protein samples (50 μg/lane) were separated by SDS-polyacrylamide gel electrophoresis. The transferred membranes were incubated with anti-phospho-Lyn, anti-phospho-Akt, anti-phospho ERK, anti-Lyn, anti-Mcl-1, and anti-procaspase 3 or anti-procaspase 9 antibodies and then developed using the enhanced chemiluminescence detection system (Amersham Pharmacia Biotech). The same blot was stripped and reprobed with anti-Lyn, anti-Akt or anti-ERK2 antibodies for use as an internal control.

### NF-κB p65 transcription factor assay

The DNA-binding activity of NF-κB was assessed using transcription factor kits for NF-κB p65 (Pierce, Rockford, IL, USA) according to the manufacturer’s instructions. DNA-binding specificity was evaluated using wild type or mutant NF-κB oligonucleotides. Chemiluminescent detection was performed using a luminometer.

### Two-dimensional electrophoresis (2DE) and MALDI-TOF/TOF

Neutrophils were incubated in the absence or presence of 10 μg/ml DP for 24 h, and the supernatant was then collected after centrifugation. Supernatant concentration was determined according to the Millipore protocols provided with the filters and the Amicon Ultra 15 was centrifuged. Aliquots in sample buffer (7 M urea, 2 M thiourea, 4.5% CHAPS, 100 mM DTE, 40 mM Tris, pH 8.8) were then applied to immobilized pH 3–10 nonlinear gradient strips (Amersham Biosciences, Uppsala, Sweden), after which isoelectricfocusing was performed at 80,000 Vh. The second dimension subsequently then analyzed on 9–16% linear gradient polyacrylamide gels at a constant voltage of 40 mA per gel for approximately 5 h. Protein spots were excised from gels with a sterile scalpel and placed into Eppendorf tubes, after which proteins were digested using trypsin (Promega, Madison, WI, USA). For MALDI-TOF/TOF MS analysis, samples were applied to the R2, R3 column and eluted with cyano-4-hydroxycinamic acid (CHCA) (Sigma, St. Louis, MO, USA) dissolved in 70% acetonitrile and 0.1% TFA before MALDI-TOF/TOF MS analysis. Next, mass spectra were acquired on a 4800 Proteomics Analyzer (Applied Biosystems) operated in MS and MS/MS modes. Peptide fragmentation in MS/MS mode was conducted by collision-induced dissociation (CID). For MS analysis, the 800–4000 *m/z* mass range was used with 1000 shots per spectrum. A maximum of 15 precursors with a minimum S/N of 50 were selected for MS/MS analysis. The MS/MS spectra were searched against the NCBInr human database (NCBInr 20120310) using the MASCOT algorithm (Matrix Science, Boston, MA, USA) for peptide and protein identification.

### Production of recombinant S100A8 and S100A9 proteins

Total RNA of human neutrophils was extracted using TRIzol reagent (Life Technologies Inc.) and first strand cDNA was synthesized with AccuPower RT PreMix (Bioneer, Daejeon, Korea). Primers S100A8-1 (5'–ttccatatgatgttgaccgagctggagaa), S100A8-2 (5'-ccgctcgagctactctttgtggctttctt), S100A9-1 (5'-ttccatatgatgacttgcaaaatgtcgca), and S100A9-2 (5'-ccgctcgagactgtggtcttagggggtgc) were used for cDNA synthesis of S100A8 and S100A9. Double-stranded cDNA of human s100A8 and S100A9 was synthesized using polymerase chain reaction (94°C 30 sec, 60°C 30 sec, 72°C 50 sec, 30 cycles) and subsequently cloned into pET28 expression vector (Merck Millipore, Darmstadt, Germany). Recombinant S100A8 and S100A9 expressions were induced with 1 mM isopropyl β-D-thiogalactoside in *E*. *coli* BL21 (DE3, Merck Millipore) for 4 h and 16 h at 37°C, respectively. Thereafter, the bacteria were centrifuged at 5000 *g* for 10 min and the pellet was lysed in BugBuster Protein Extraction reagent (Merck Millipore). Next, the lysate was centrifuged and the supernatant was collected. Recombinant His-Tag S100A8 and S100A9 were purified using a nickel column and the purified proteins were verified by SDS-PAGE and western blotting (S1 Fig).

### Flow cytometry

After treatment with DP, human neutrophils were harvested and washed twice with PBS. The cells were then fixed with 100 μl of 0.37% paraformaldehyde solution for 15 min at room temperature. Following removal of the fixing solution, the cells were added to 100 μl of 0.2% Triton X-100 in PBS and incubated for 3 min. Next, the cells were washed twice with PBS buffer containing 0.5% BSA, after which non-specific antibody binding was reduced by incubating the cells with normal rabbit IgG. The cells were subsequently separated into new tubes, to which PBS buffer containing anti-S100A8 and anti-S100A9 antibodies was added. Baseline fluorescence was obtained by incubation with normal mouse IgG instead of anti-S100 protein antibodies. After washing three times, the cells were incubated at 4°C for 30 min with FITC-conjugated goat anti-mouse IgG (Molecular Probes; Eugene, OR, USA). Finally, the cells were washed and analyzed on a FACSort cytofluorimeter (Becton Dickinson). For each experiment, 10,000 events were collected. The mean intensity of untreated cells was considered 100%. Alteration of intracellular S100A8 and S100A9 expression after DP treatment was evaluated as the mean intensity of DP-treated cells/the mean intensity of untreated cells × 100.

### Enzyme-linked immunosorbent assay (ELISA)

96-well plates were coated with 100 μl/well of anti-S100A8 and anti-S100A9 antibodies in 0.1 M carbonate buffer and incubated overnight at 4°C. The plates were then washed with PBS solution with 0.05% Tween-20 and blocked with PBS solution with 5% bovine serum albumin (BSA) for 30 min at room temperature. Next, BALF and serum were added to the plates and incubated for 2 h at room temperature. The plates were then washed three times and incubated with anti-S100A8 and anti-S100A9 antibodies for 2 h at room temperature. The plates were then washed three times and incubated with substrate solution. Finally, the reaction was blocked by adding stop buffer and the absorbance was read at 450 nm.

### Collection of bronchoalveolar lavage fluid (BALF) and cell count

Following local anesthesia with lignocaine, sterile phosphate-buffered saline (PBS) (5×20 mL) was administered, after which the fluid was gently aspirated, pooled and collected in a tube. Nucleated cells in BALF and blood were counted using a Neubauer hemocytometer. Differential cell counts were performed from cytospin slides.

### Statistical analysis

Data were expressed as the means ± SD. Statistical differences were analyzed using a paired t-test for a two-group comparison and one-way ANOVA for comparison of more than two groups. The SPSS statistical software package (Version 10.0, Chicago, IL) was used for statistical analysis. A *p* value < 0.05 was deemed statistically significant. Spearman’s correlation analysis was used to determine the correlation of the expression levels of S100 proteins and neutrophil counts or physiological function results [FEV1(%) and FEV1/FVC (%)].

## Results

### DP delays constitutive neutrophil apoptosis in normal subjects

**Fig 1:**
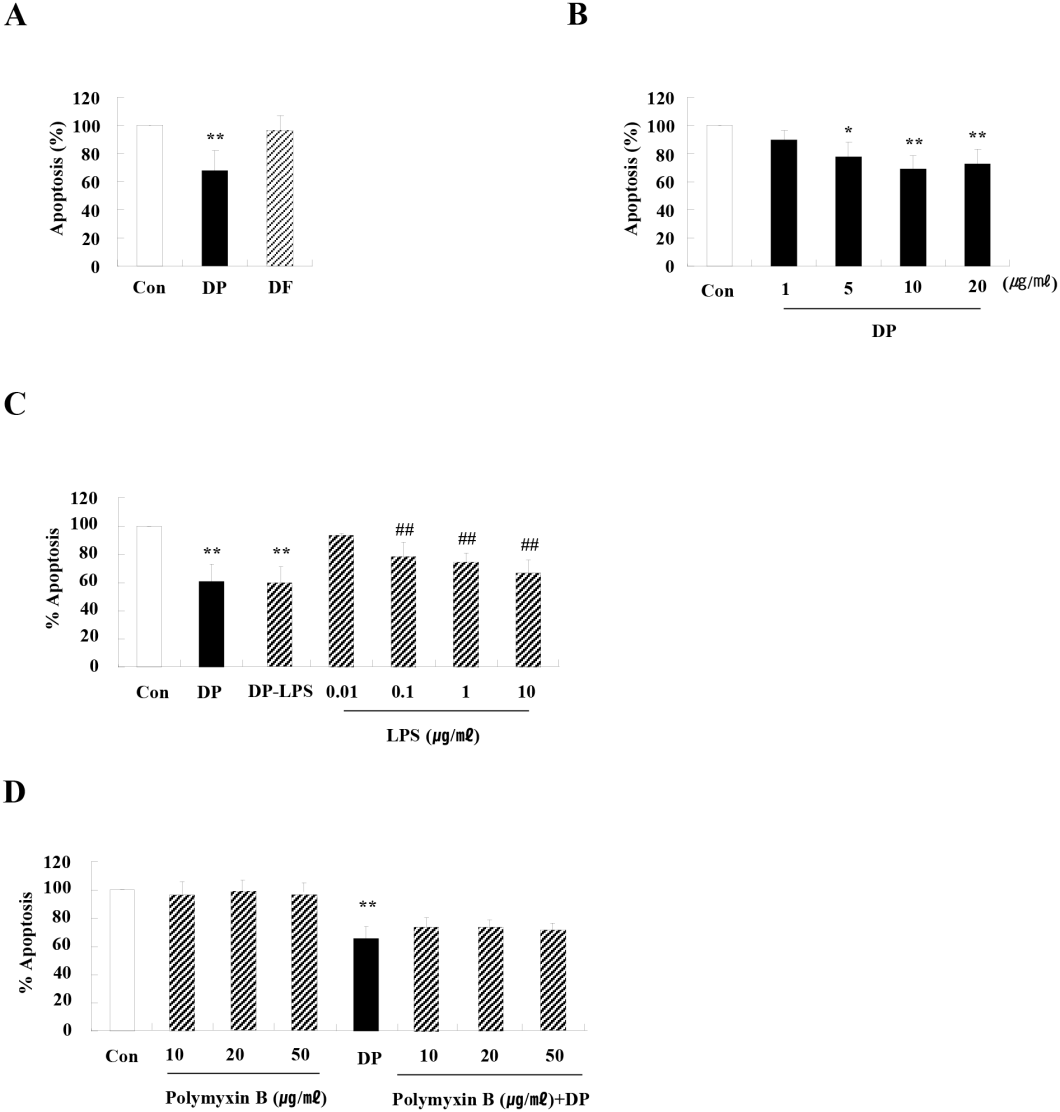
DP delays constitutive neutrophil apoptosis in normal subjects. (A) Neutrophils were isolated from the peripheral blood of normal subjects (n = 59) and then incubated for 24 h in the absence (Con) and presence of DP (10 μg/mL) and DF (10 μg/mL). (B) Neutrophils (n = 4) were incubated for 24 h in the absence (Con) and presence of DP in the indicated concentration. (C) Neutrophils (n = 3) were incubated for 24 h in the absence (Con) and presence of DP, DP-LPS [LPS-removed DP using Endo Trap Red (Lonza, MD)], or LPS. (D) Normal neutrophils (n = 3) were pre-treated for 1 h with and without polymyxin B in the indicated concentration, after which the cells were incubated for 24 h in the absence and presence of DP (10 μg/ml). Apoptosis was analyzed by measuring the binding of annexin V-FITC and PI. Data are expressed as the means ± SD and are presented relative to the control, which was set at 100%. *p < 0.05 and **p < 0.01 indicate a significant difference between the control and stimulator-treated groups.

To investigate the effects of HDM on neutrophil apoptosis, we first evaluated whether major allergens of HDM, DP and DF alter the regulation of neutrophil apoptosis. DP blocked neutrophil apoptosis in a dose-dependent manner, and DF had no effect on apoptosis (Fig 1A and 1B). DP inhibited spontaneous apoptosis of neutrophils in a time-dependent manner, and DP is not effective on early action but late action (data not shown). The anti-apoptotic effect of DP was not associated with LPS (Fig 1C and 1D).

### DP induces inhibition of neutrophil apoptosis via activation of TLR4, Lyn, PI3K, Akt, ERK and NF-κB, and suppression of the caspase9/3 pathway

**Fig 2:**
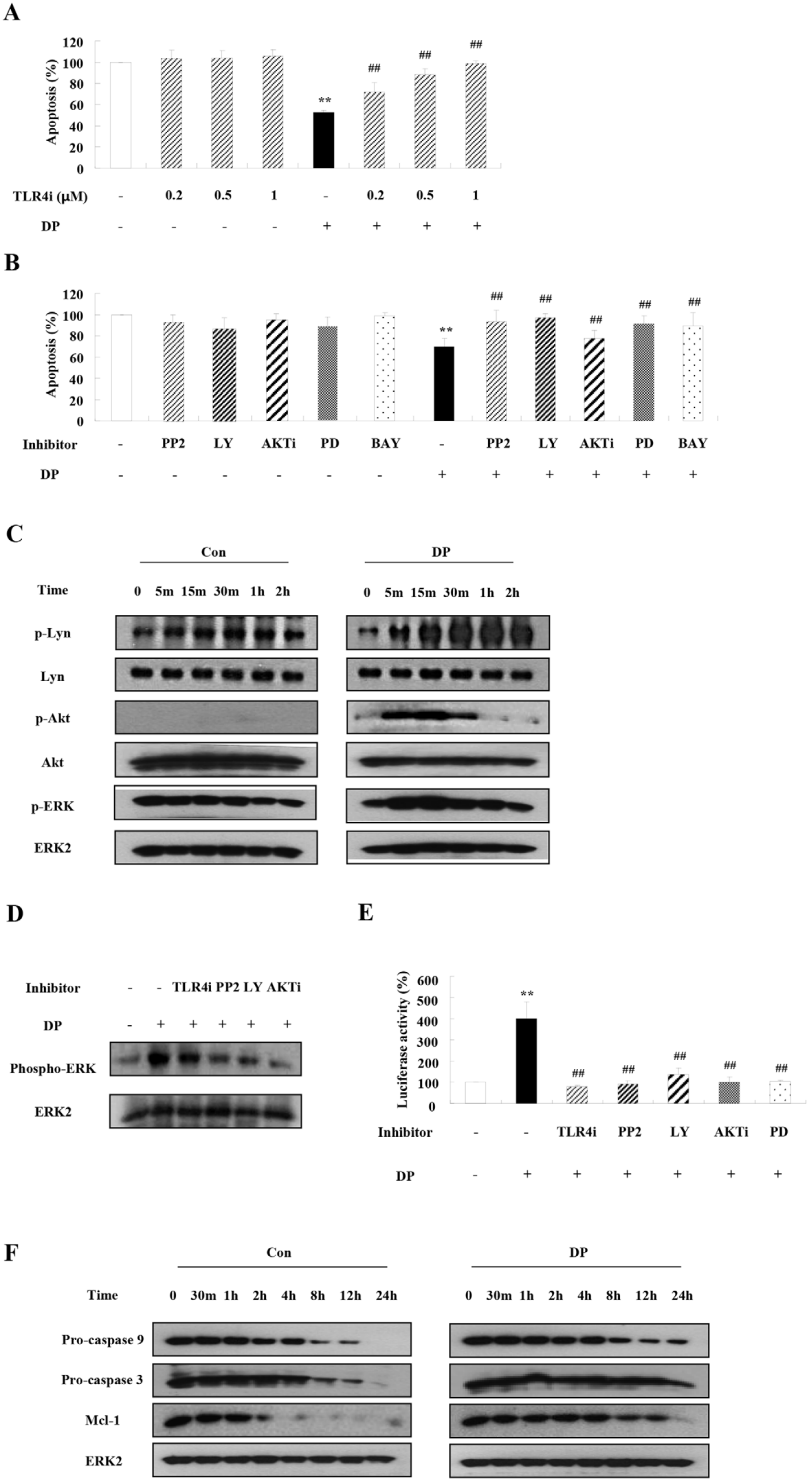
DP induces the inhibition of neutrophil apoptosis via activation of TLR4, Lyn, PI3K, Akt, ERK and NF-κB, and suppression of the caspase9/3 pathway. (A-B) Normal neutrophils (3<n<7) were pre-treated for 1 h with and without TLR4i in the indicated concentration (A) or 10 μM PP2, 10 μM Ly294002 (LY), 10 μM AKTi, 10 μM PD98059 (PD) and 10 μM BAY-11-7085 (BAY) (B), after which the cells were incubated for 24 h in the absence and presence of DP (10 μg/ml). Apoptosis was analyzed by measuring the binding of annexin V-FITC and PI. Data are presented relative to the control, which was set at 100% of the means ± SD. **p < 0.01 indicates a significant difference between the control and DP-treated groups, and ^##^ *p* < 0.01 represents a significant difference between the DP-treated group and the inhibitor-treated group. (C) Normal blood neutrophils were incubated with DP (10 μg/ml) for the indicated time. Phosphorylation of Lyn, Akt and ERK in the lysates was detected by Western blotting. (D) Normal blood neutrophils were pre-treated for 1 h with and without 1 μM TLR4i, 10 μM PP2, 10 μM Ly294002 (LY) and 10 μM AKTi, and then incubated with DP (10 μg/ml) for 30 min. Phosphorylation of ERK in the lysates was detected by Western blotting. (E) Normal neutrophils were pre-treated for 1 h with and without 1 μM TLR4i, 10 μM PP2, 10 μM Ly294002 (LY), 10 μM AKTi, and 10 μM PD98059 (PD) and then incubated with DP (10 μg/ml) for 8 h. The nuclear fraction was extracted, and the NF-κB DNA binding activity was assessed using an transcription factor kit. **p < 0.01 indicates a significant difference between the control and DP-treated groups, and ^##^ *p* < 0.01 represents a significant difference between the DP-treated group and the inhibitor-treated groups. (F) Normal blood neutrophils were incubated with DP (10 μg/ml) for the indicated time. Procaspase 9, procaspase 3 and Mcl-1 proteins were detected by Western blotting. The membrane was stripped and reprobed with anti-ERK2 antibodies as an internal control.

As shown in Fig 2A and 2B, TLR4i, PP2, Ly294002, AKTi, PD98059, and BAY 11–7085 blocked the suppressive effects on neutrophil apoptosis induced by DP. Lyn, Akt, and ERK were phosphorylated by DP in a time-dependent manner (Fig 2C), while DP-induced ERK activation was suppressed by TLR4i, PP2, Ly294002, and AKTi (Fig 2D). In this study, DP induced NF-κB activation at 8 h as our previous report [13], and this activation was inhibited by TLR4i, PP2, Ly294002, and PD98059 (Fig 2E). The expression of procaspase 9 and procaspase 3 decreased after constitutive apoptosis began, indicating that both procaspases were cleaved. DP delayed the decreased expression of procaspase 9 and procaspase 3 (Fig 2F). The expression of Mcl-1 decreased in a time-dependent manner, while the decrease of Mcl-1 was inhibited by DP.

### Both S100A8 and S100A9 are released after DP treatment, which induces the inhibition of neutrophil apoptosis through activation of TLR4, Lyn, PI3K, Akt, ERK and NF-κB, and via suppression of the caspase9/3 pathway

**Fig 3:**
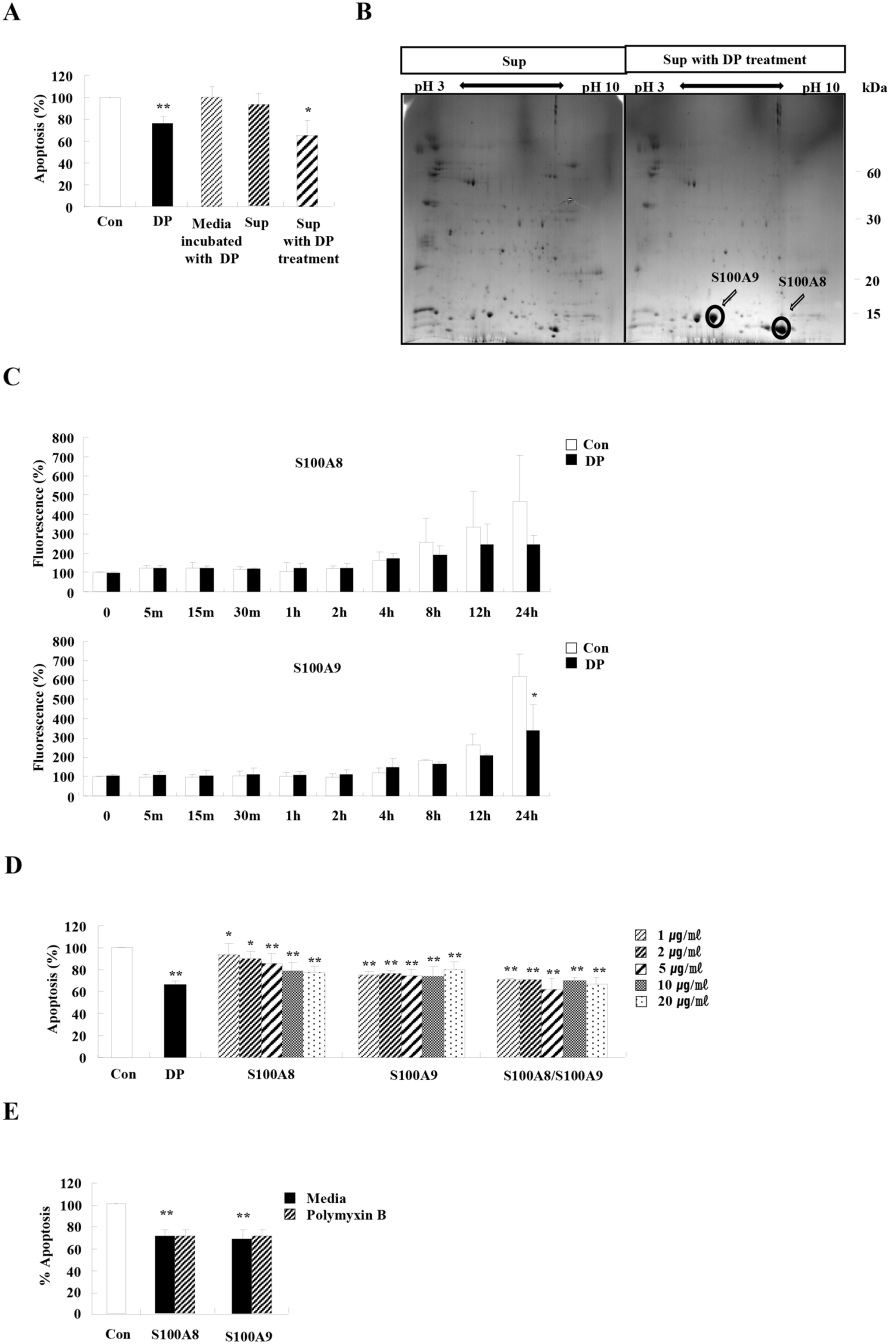
Both S100A8 and S100A9 are released after DP treatment and suppress neutrophil apoptosis. (A) Neutrophils were incubated with and without 10 μg/ml of DP for 24 h. The supernatant (Sup) was collected and added to the fresh neutrophils obtained from the peripheral blood of normal individuals (n = 9). Media were incubated with DP for 24 h in a 5% CO_2_ incubator at 37°C. The media were collected and added to the fresh neutrophils obtained from the peripheral blood of normal individuals (n = 5). Apoptosis was analyzed by measuring the binding of annexin V-FITC and PI. Data are expressed as the means ± SD and are presented relative to the control, which was set at 100%. **p < 0.01 indicates a significant difference between the control and the DP-treated group, and *p < 0.05 represents a significant difference between the control group and the Sup with DP treatment-treated group. (B) Neutrophils were incubated with and without 10 μg/ml of DP for 24 h. The supernatant (Sup) was collected and analyzed by 2DE and MALDI-TOF/TOF. (C) Neutrophils (n = 3) were incubated with 10 μg/ml of DP for the indicated time. The cells were fixed and permeabilized with 0.37% paraformaldehyde solution and 0.2% Triton X-100 solution, respectively, then incubated with anti-S100A8 or anti-S100A9 antibodies and analyzed on a FACSort cytofluorimeter. The mean intensity of untreated cells was considered 100%. Alteration of intracellular S100A8 and S100A9 expression after DP treatment was evaluated as the mean intensity of DP-treated cells/the mean intensity of untreated cells × 100. (D) Neutrophils (3<n<6) were incubated for 24 h in the absence (Con) and presence of S100A8 and S100A9 (10 μg/mL) in the indicated concentration. (E) Normal neutrophils (n = 4) were pre-treated for 1 h with and without 50 μg/ml polymyxin B after which the cells were incubated for 24 h in the absence and presence of S100A8 or S100A9DP (10 μg/ml). Apoptosis was analyzed by measuring the binding of annexin V-FITC and PI. Data are expressed as the means ± SD and are presented relative to the control, which was set at 100%. *p < 0.05 and **p < 0.01 indicate a significant difference between the control and stimulator-treated groups.

As shown in Fig 3A, supernatant collected after DP treatment effectively inhibited neutrophil apoptosis. To identify unknown proteins of supernatant associated with the anti-apoptotic effect, we conducted 2DE and MALDI-TOF/TOF, which revealed that the expression of S100A8 and S100A9 increased after DP treatment (S100A9: sequence coverage 31%, number of matched peptide: 3, a representative peptide: R.LTWASHEK.M, score 46) (S100A8: sequence coverage 33%, number of matched peptide: 3, a representative peptide: K.ALNSIIDVYHK.Y, score 64) (Fig 3B). Intracellular S100A8 and S100A9 proteins increased after spontaneous apoptosis began, but this increase lessened in response to DP treatment, indicating that DP induced secretion of intracellular S100A8 and S100A9 (Fig 3C). This increase of S100A8 and S100A9 led us to examine the direct anti-apoptotic effect of S100A8 and S100A9. We produced recombinant S100A8 and S100A9 proteins, and both proteins suppressed neutrophil apoptosis (Fig 3D). The anti-apoptotic effects of S100A8 and S100A9 were not associated with LPS (Fig 3E).

### S100A8 and S100A9 trigger the suppression of neutrophil apoptosis through activation of TLR4, Lyn, PI3K, Akt, ERK and NF-κB, and via suppression of the caspase9/3 pathway

**Fig 4:**
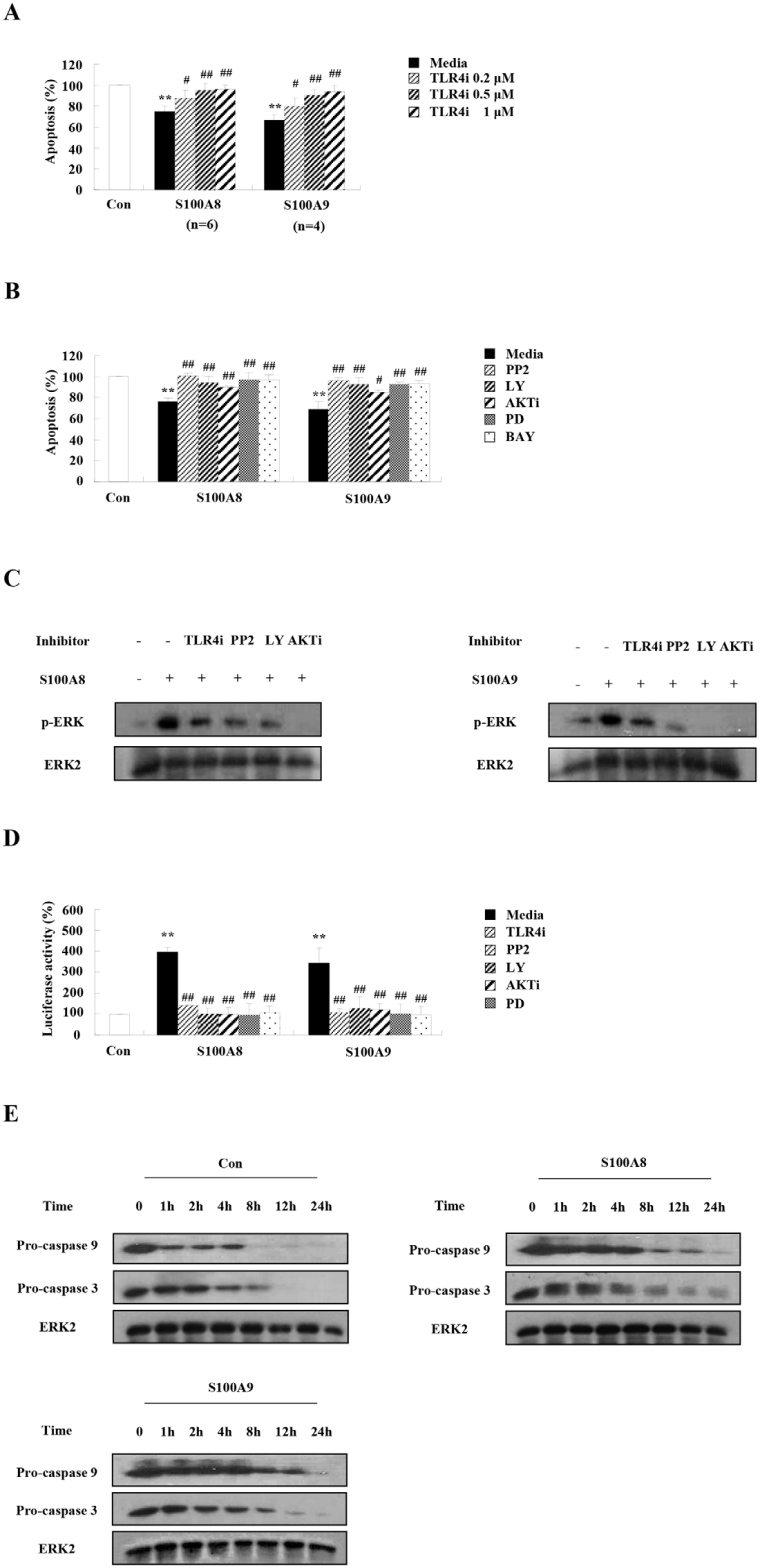
S100A8 and S100A9 trigger the suppression of neutrophil apoptosis through activation of TLR4, Lyn, PI3K, Akt, ERK and NF-κB, and via suppression of the caspase9/3 pathway. (A-B) Normal neutrophils (4<n<6) were pre-treated for 1 h with and without TLR4i in the indicated concentration (A) or 10 μM PP2, 10 μM Ly294002 (LY), 10 μM AKTi, 10 μM PD98059 (PD) and 10 μM BAY-11-7085 (BAY) (B), after which the cells were incubated for 24 h in the absence and presence of S100A8 and S100A9 (10 μg/ml). Apoptosis was analyzed by measuring the binding of annexin V-FITC and PI Data are expressed as the means ± SD and are presented relative to the control, which was set at 100%. **p < 0.01 indicates a significant difference between the control and DP-treated groups, and ^#^ *p* < 0.05 and ^##^ *p* < 0.01 represent a significant difference between the DP-treated group and the inhibitor-treated group. (C) Normal blood neutrophils were pre-treated for 1 h with and without 1 μM TLR4i, 10 μM PP2, 10 μM Ly294002 (LY) and 10 μM AKTi, and then incubated with S100A8 and S100A9 (10 μg/ml) for 30 min. Phosphorylation of ERK in the lysates was detected by Western blotting. (D) Normal neutrophils were pre-treated for 1 h with and without 1 μM TLR4i, 10 μM PP2, 10 μM Ly294002 (LY), 10 μM AKTi, and 10 μM PD98059 (PD) and then incubated with S100A8 and S100A9 (10 μg/ml) for 8 h. The nuclear fraction was extracted, after which the NF-κB DNA binding activity was assessed using an transcription factor kit. **p < 0.01 indicates a significant difference between the control and DP-treated groups, and ^##^ *p* < 0.01 represents a significant difference between the DP-treated group and the inhibitor-treated group. (E) Normal blood neutrophils were incubated with S100A8 and S100A9 (10 μg/ml) for the indicated time. Procaspase 9 and procaspase 3 were detected by Western blotting. The membrane was stripped and reprobed with anti-ERK2 antibodies as an internal control.

Because S100A8 and S100A9 is secreted by neutrophils under the influence of DP and has an inhibitory effect on neutrophil apoptosis, we investigated how they act as anti-apoptotic proteins comparable to the DP-mediated signaling mechanism. TLR4i inhibited the anti-apoptotic effects of S100A8 and S100A9 in a dose-dependent manner (Fig 4A). DP, S100A8 and S100A9 require TLR4 to induce their anti-apoptotic mechanism. Specific signal inhibitors such as PP2, Ly294002, AKTi, PD98059, and BAY-11-7085 significantly blocked the inhibitory effects of S100A8 and S100A9 (Fig 4B). ERK activation due to S100A8 and S100A9 was blocked by TLR4i, PP2, Ly294002, and AKTi (Fig 4C). The inhibition of NF-κB activation by PP2, Ly294002, and PD98059 indicates that Lyn, PI3K, Akt, and ERK are upstream molecules of NF-κB activation due to S100A8 and S100A9 (Fig 4D). S100A8 and S100A9 also delayed the activation of the pro-apoptotic proteins, procaspase 9 and procaspase 3 (Fig 4E).

### DP, S100A8, and S100A9 have anti-apoptotic effects in asthmatic neutrophils

**Fig 5:**
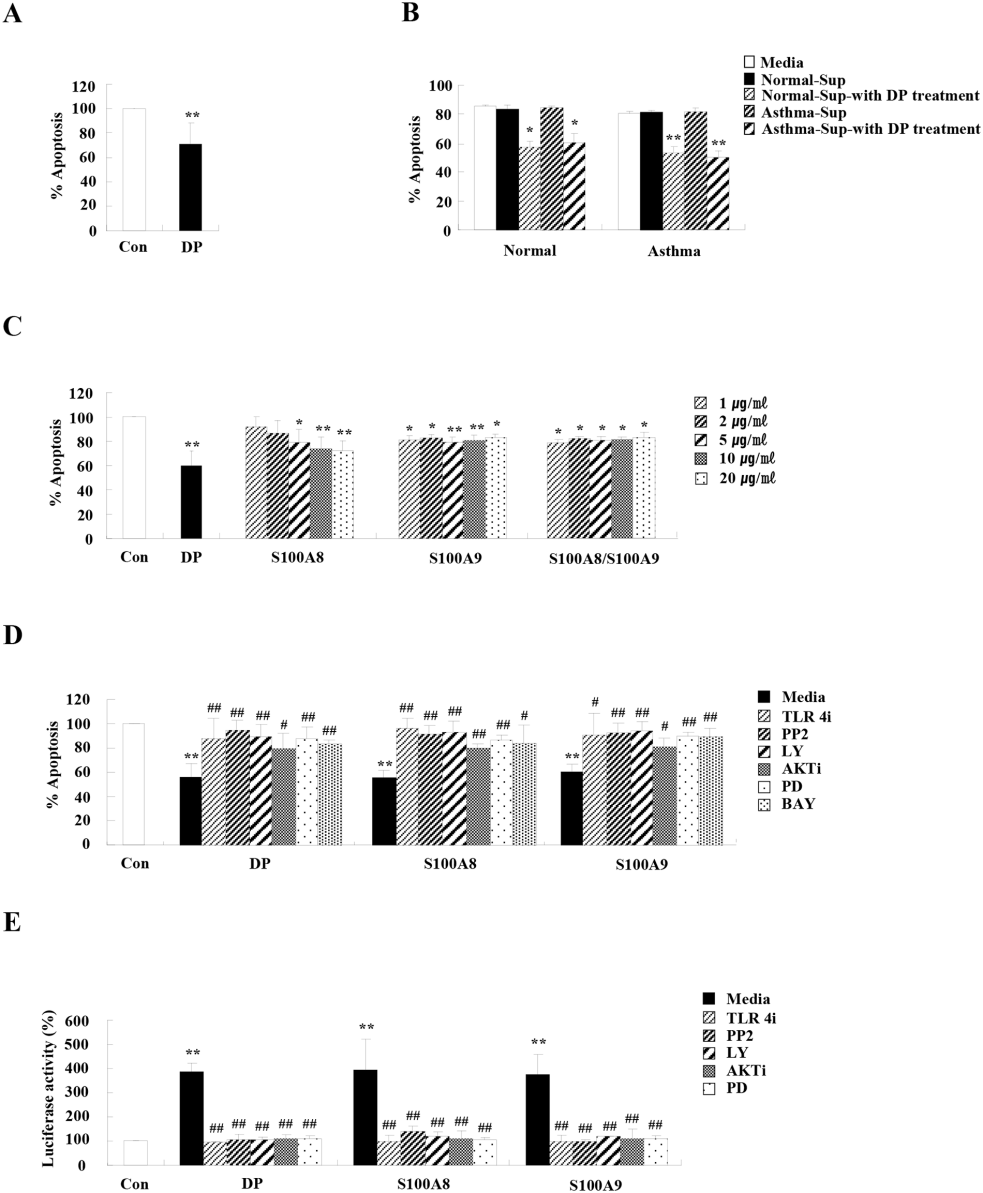
DP, S100A8, and S100A9 have anti-apoptotic effects in asthmatic neutrophils. (A-E) (A) Neutrophils were isolated from the peripheral blood of asthmatic subjects (n = 54). The cells were incubated for 24 h in the absence (Con) and presence of DP (10 μg/mL) (B) Normal and asthmatic neutrophils were incubated with and without 10 μg/ml of DP for 24 h. The supernatant (Sup) was collected and added to the fresh neutrophils isolated from the peripheral blood of normal and asthmatic subjects (n = 9). (C) Asthmatic neutrophils (n = 3) were incubated for 24 h in the absence (Con) and presence of S100A8 and S100A9 in the indicated dose. (D) Asthmatic neutrophils (n = 3) were pre-treated for 1 h with and without 1 μM TLR4i, 10 μM PP2, 10 μM Ly294002 (LY), 10 μM AKTi, 10 μM PD98059 (PD) and 10 μM BAY-11-7085 (BAY), after which the cells were incubated for 24 h in the absence and presence of S100A8 and S100A9 (10 μg/ml). Apoptosis was analyzed by measuring the binding of annexin V-FITC and PI. Data are expressed as the means ± SD and are presented relative to the control, which was set at 100%. (E) Asthmatic neutrophils were pre-treated for 1 h with and without 1 μM TLR4i, 10 μM PP2, 10 μM Ly294002 (LY), 10 μM AKTi, and 10 μM PD98059 (PD) and then incubated with S100A8 and S100A9 (10 μg/ml) for 8 h. The nuclear fraction was extracted, and the NF-κB DNA binding activity was assessed using an transcription factor kit. *p < 0.05 and **p < 0.01 indicate a significant difference between the control and stimulator-treated groups, and ^#^ *p* < 0.05 and ^##^ *p* < 0.01 represent a significant difference between the stimulator-treated and inhibitor-treated groups.

Because DP prolongs neutrophil survival of normal subjects, we examined whether DP alters the constitutive apoptosis of asthmatic neutrophils comparable to normal neutrophil apoptosis. First, we investigated correlation of neutrophil number with the results of a physiological function test. Neutrophil count in the blood and BALF of asthmatic subjects was negatively correlated with FEV1(%) and FEV1/FVC(%), indicating that neutrophils are associated with asthma pathogenesis and severity (*p*<0.01) (S2 Fig). As shown in Fig 5A, DP inhibited neutrophil apoptosis of asthmatics comparable to the effect of DP on normal neutrophil apoptosis. Der p 1, Der p 2, E64, and aprotinin, had no effect on asthmatic neutrophil apoptosis (S3 Fig). Supernatant from asthmatic neutrophils after DP treatment suppressed the apoptosis of normal and asthmatic neutrophils, and supernatants from normal neutrophils also effectively suppressed the apoptosis of normal and asthmatic individuals (Fig 5B). S100A8 and S100A9 suppressed the apoptosis of asthmatic neutrophils comparable to normal neutrophil apoptosis (Fig 5C). To further investigate the intracellular signaling pathway of DP, S100A8, and S100A9, we evaluated alteration of asthmatic neutrophil apoptosis of DP, S100A8, and S100A9 using signal specific inhibitors. TLR4i, PP2, Ly294002, AKTi, PD98059, and BAY 11–7085 reversed the inhibitory effects on neutrophil apoptosis induced by DP, S100A8, and S100A9 (Fig 5D). DP, S100A8, and S100A9 induced NF-κB activation, and this activation was suppressed by TLR4i, PP2, Ly294002, AKTi, and PD98059 (Fig 5E).

### Elevated S100A8 level in BALF is associated with increased BALF neutrophils and decreased physiological function

**Fig 6:**
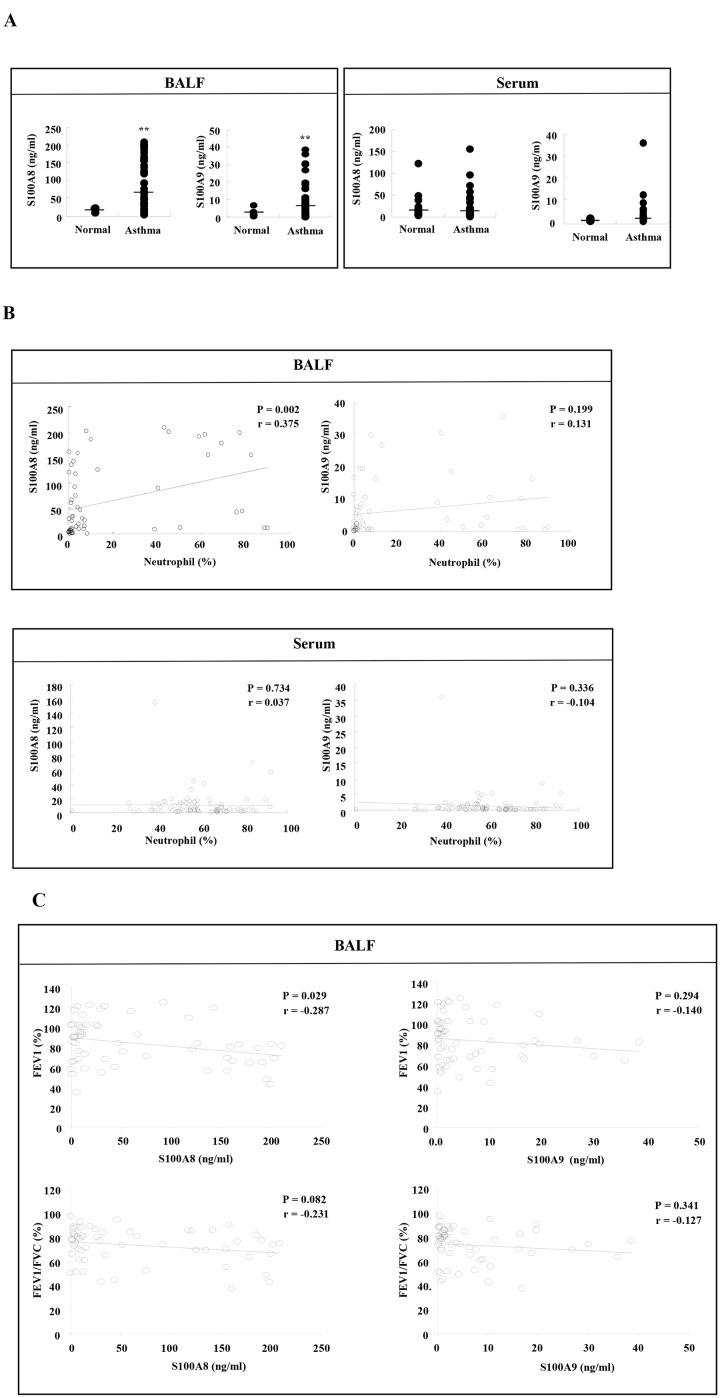
Elevated S100A8 level in BALF is associated with an increase of BALF neutrophils and a decrease of physiological function. (A) The concentration of S100A8 and S100A9 in BALF and serum of normal (6<n<26) and asthmatic subjects (61<n<94) was measured by ELISA as described in the Materials and Method section. **p < 0.01 indicates a significant difference between the normal and asthmatic groups. (B) Linear regression represents the correlation between S100A8 or S100A9 and the number of neutrophils (%) in asthmatic subjects. (C) Linear regression represents the correlation between S100A8 or S100A9 in BALF and FEV1 (%) or FEV1/FVC (%).

Since DP increases the secretion of S100A8 and S100A9, which have inhibitory effects on neutrophil apoptosis *in vitro*, we investigated the clinical relevance of the above findings in asthma subjects. Both S100A8 and S100A9 proteins were significantly elevated in BALF (*p*<0.01), but not in serum (Fig 6A). These findings indicate that increases of S100A8 and S100A9 are not associated with systemic phenomena, but rather with local phenomena in lung tissue. The number of asthmatic BALF neutrophils was significantly correlated with the concentration of S100A8 (*p*<0.01), whereas there was no correlation between neutrophil number and S100A9 expression (Fig 6B). Additionally, there was no correlation between serum S100A8 or S100A9 and the number of asthmatic BALF neutrophils (Fig 6B). S100A8 expression in BALF is negatively correlated with FEV1(%), despite the expression not being associated with FEV1/FVC(%). These data indicate that S100A8 expression of BALF is related to neutrophil apoptosis and asthma severity (Fig 6C).

## Discussion

In this study, we have shown than TLR4 functions as a pivotal receptor in the anti-apoptotic mechanism of DP in normal and asthmatic neutrophils (Figs 2 and 5). DP transduces direct anti-apoptotic signaling through TLR4, and S100A8 and S100A9 secreted by DP induces anti-apoptotic effects through TLR4. Although TLR4 signaling acts as an anti-inflammatory mechanism in acute lung inflammation induced by hyaluronan, recent reports have demonstrated the importance of TLR4 in HDM-mediated asthma and we reported the association of TLR4 and DP in allergic rhinitis [3, 16–19]. DP induces asthmatic features via TLR4 in airway structural cells and airway inflammation in the absence of TLR4 of hemopoietic cells [3]. In sharp contrast, our results suggest that TLR4 is essential to protection from apoptosis of neutrophils, which are differentiated from hemopoietic cells. Different results may be caused by differences between mouse and human experimental models, and by the fact that the mouse used in the previous study is an eosinophilic asthmatic animal model. Although a recent report demonstrated that HDM is mediated through TLR2 as well as TLR4, regulation of neutrophil apoptosis due to DP is not altered by TLR2-blocking antibodies (S4 Fig) [20]. Survival factors such as GM-CSF, G-CSF, IL-8 and TNF-α transduce anti-apoptotic signals through the PI3K/Akt pathway and the PKC pathway, as well as regulation of caspase and Bcl-2 family proteins [11, 12]. DP, S100A8, and S100A9 activate Lyn, PI3K, Akt, ERK and NF-κB, and suppress caspase 9/3 pathway in normal and asthmatic neutrophils (Figs 2, 4 and 5).

DP and DF are prevalent among HDMs that produce at least 23 groups of allergens and are immunologically cross-reactive [21, 22]. Additionally, proteins between DP and DF have 15–20% amino acid sequence disparity. As shown in Fig 1ADP and DF have different effects on neutrophil apoptosis, which indicates that the difference in HDM species evokes a greater variety of pathogenic mechanisms of allergic inflammation than expected. Because exposure of asthmatic subjects to HDM is important to the diagnosis and therapy of asthma, we classified atopic (DP and/or DF-specific IgE positive) and non-atopic asthmatics. However, anti-apoptotic effects of DP and concentrations of S100A8 and S100A9 in BALF and serum did not differ between atopic and non-atopic asthma (S5 Fig). In addition, there was no age dependency observed in our results, although the average age of normal and asthmatic individuals differed (Table 1). DP contains proteases, lipid-binding proteins, β-glucan, chitin, and other unknown materials [23]. Der p 1 is a cysteine protease and triggers its role via PAR [24]. Der p 2 is a MD-like protein. The association between Der p 2 and TLR4 is controversial. Der p 2 induces allergic asthma by direct TLR4 binding or by TLR4 binding after interacting with LPS [18,25]. However, a recent report demonstrated that Th2-biased response induced by Der p 2 is independent on functional TLR4 [26]. As shown in S3 Fig, the protease inhibitors of Der p 1 and Der p 2 had no effect on neutrophil apoptosis of normal and asthmatic individuals. Although Der p 7 may mediate TLR4 signaling, Der p 7 has no effect on apoptosis [27] (S6 Fig). Therefore, we think that a novel anti-apoptotic factor binding to TLR4, which is not homologous to DF proteins, exists in DP. The exact factor within DP involved in this process was not identified in this study; accordingly, further studies are being conducted to gain further insight and more clearly define the mechanisms involved in this reaction.

Because an anti-apoptotic factor stimulates cytokine secretion and the released proteins play a role in survival, we examined the secretory proteome induced by DP and identified S100A8 and S100A9. Both S100A8 and S100A9 are abundant and their constitutive expression is restricted to neutrophils [28]. S100A8 and S100A9 are important pathogenic mediators in severe asthma because airway resident cells express TLR4, which binds to S100A8 and S100A9, and because both proteins induce pro-inflammatory responses [29–31]. Our results show that S100A8 and S100A9 secreted by neutrophils after DP stimulation have an anti-apoptotic effect on the neutrophils, suggesting they act in an autocrine/paracrine fashion (Fig 3). Neutrophil apoptosis is regulated by cell concentrations via S100A8 and S100A9, and the inhibitory effect of S100A8 or S100A9 is very low (5–13%) compared to our results (30–60%) [32]. Therefore, this report may not clearly demonstrate the direct effects of S100A8 and S100A9 on neutrophil apoptosis. In addition, our results show that S100A8 and S100A9 suppress asthmatic neutrophil apoptosis as well as normal apoptosis (Figs 3 and 5). S100A8 expression is positively correlated to the number of BALF neutrophils and negatively correlated to FEV1(%) (Fig 6). In contrast to the effects of S100A8 in neutrophils, the concentration of S100A9 is not associated with clinical features, including the number of BALF neutrophils and physiological function results. This may occur because S100A9 shows lower expression in asthmatic subjects than S100A8 (Fig 6A).

**Fig 7:**
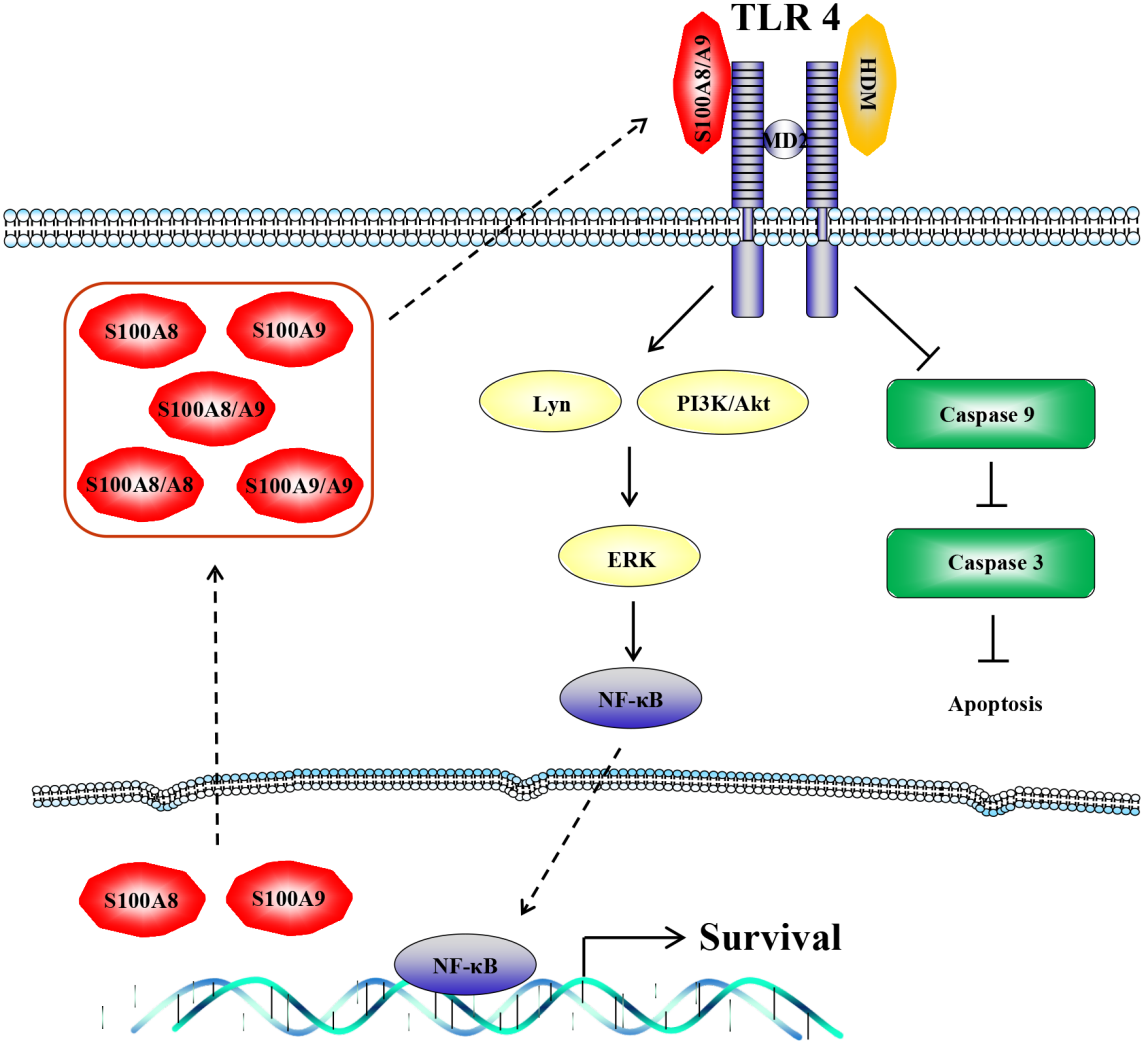
The proposed anti-apoptotic signaling pathway induced by DP in normal and asthmatic neutrophils. Anti-apoptotic signaling due to DP, S!00A8, and S100A9 involves TLR4, Lyn, PI3K, Akt, ERK and NF-κB, and is associated with suppression of procaspase 3 and procaspase 9 cleavage.

In summary, DP delays the constitutive apoptosis and induces secretion of S100A8 and S100A9 in normal and asthmatic neutrophils. TLR4 functions as a common receptor in anti-apoptotic signaling of DP, S100A8 and S100A9 (Fig 7). These findings will help unveil regulation of normal neutrophil survival and link house dust mites with asthma pathogenesis.

## Supporting Information

S1 Fig Production of recombinant S100A8 and S100A9 proteins. Both recombinant S100A8 and S100A9 proteins are produced as the materials and methods section. The purified proteins were verified by SDS-PAGE (A) and western blotting (B).
(TIF)

S2 Fig Asthmatic neutrophils in BALF and serum is negatively associated with FEV1(%) and FEV1/FVC(%). Linear regression represents the correlation between neutrophils in BALF and serum and FEV1(%) or FEV1/FVC(%).
(TIF)

S3 Fig Anti-apoptotic effect of DP in asthmatic neutrophils is not affected by Der p 1, Der p 2 and protease inhibitors. Normal, (A) and asthmatic (B) neutrophils (8<n<29), were incubated for 24 h with and without DP (10 μg/ml), Der p 1 (10 μg/ml) and Der p 2 (10 μg/ml) (left panel) or were pretreated in the absence and presence of E64 (50 μg/ml) and aprotinin (Ap) (50 μg/ml) for 1 h, after which the cells were incubated for 24 h in the absence and presence of DP (10 μg/ml) (right panel). Apoptosis was analyzed by measuring the binding of annexin V-FITC and PI. Data are expressed as the means ± SD and are presented relative to the control, which was set at 100%. *p < 0.05 and **p < 0.01 indicate a significant difference between the control and DP-treated groups.
(TIF)

S4 Fig Anti-apoptotic effect of DP in asthmatic neutrophils is not affected by blocking antibody against TLR2. Normal neutrophils (n = 3) were pretreated in the absence and presence of anti-TLR2 blocking antibodies in the indicated concentration for 1 h, after which the cells were incubated for 24 h in the absence and presence of DP (10 μg/ml). Apoptosis was analyzed by measuring the binding of annexin V-FITC and PI. Data are expressed as the means ± SD and are presented relative to the control, which was set at 100%. **p < 0.01 indicates a significant difference between the control and DP-treated groups.
(TIF)

S5 Fig Anti-apoptotic effect of DP and concentrations of S100A8 and S100A9 in BALF and serum are not different between atopic and non-atopic asthma. (A-B) Data are presented by classifying the results from Fig 5A (A) and from Fig 6A (B), depending on non-atopic and atopic asthma.
(TIF)

S6 Fig Der p 7 has no effect on spontaneous apoptosis of normal neutrophils. Normal neutrophils (n = 7) were incubated for 24 h with and without Der p 7 (10 μg/ml). Apoptosis was analyzed by measuring the binding of annexin V-FITC and PI. Data are expressed as the means ± SD and are presented relative to the control, which was set at 100%.
(TIF)
