# Supplementary figures and images for: House Dust Mite Allergen Regulates Constitutive Apoptosis of Normal and Asthmatic Neutrophils via Toll-Like Receptor 4
Source: PLoS One. 2015 May 14;10(5):e0125983. doi: 10.1371/journal.pone.0125983 (PMC4431853; doi:10.1371/journal.pone.0125983)

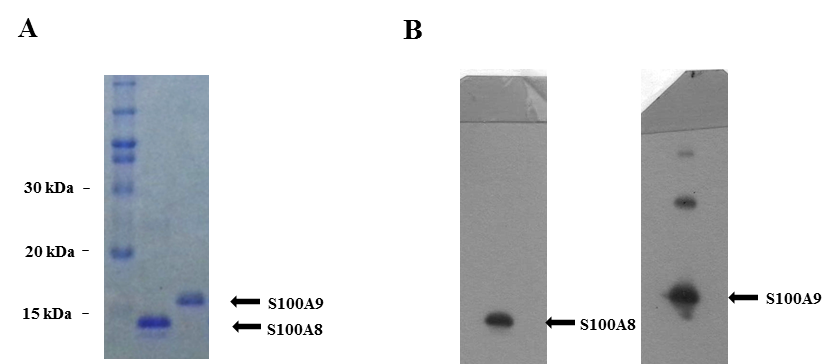

Supplement: S1 Fig — Both recombinant S100A8 and S100A9 proteins are produced as the materials and methods section. The purified proteins were verified by SDS-PAGE (A) and western blotting (B). (TIF) [file pone.0125983.s001.tif]

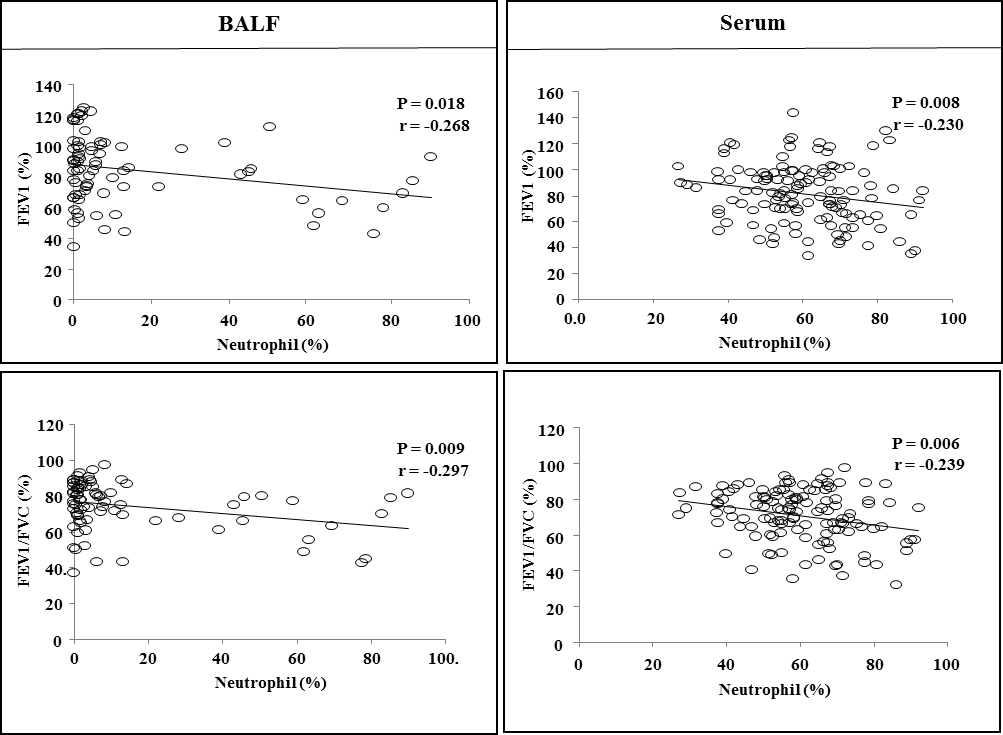

Supplement: S2 Fig — Linear regression represents the correlation between neutrophils in BALF and serum and FEV1(%) or FEV1/FVC(%). (TIF) [file pone.0125983.s002.tif]

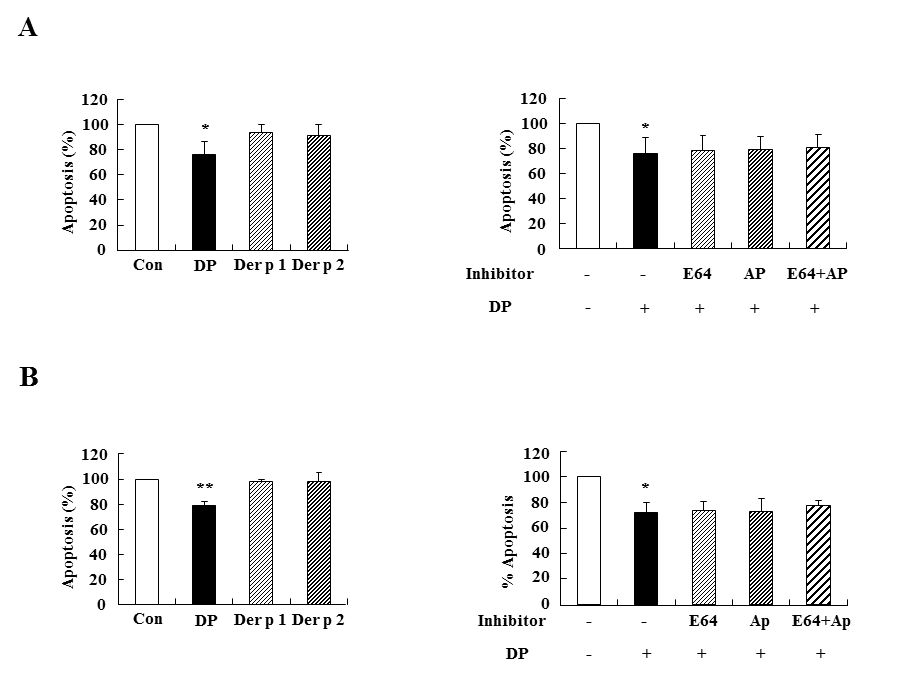

Supplement: S3 Fig — Normal, (A) and asthmatic (B) neutrophils (8<n<29), were incubated for 24 h with and without DP (10 μg/ml), Der p 1 (10 μg/ml) and Der p 2 (10 μg/ml) (left panel) or were pretreated in the absence and presence of E64 (50 μg/ml) and aprotinin (Ap) (50 μg/ml) for 1 h, after which the cells were incubated for 24 h in the absence and presence of DP (10 μg/ml) (right panel). Apoptosis was analyzed by measuring the binding of annexin V-FITC and PI. Data are expressed as the means ± SD and are presented relative to the control, which was set at 100%. *p < 0.05 and **p < 0.01 indicate a significant difference between the control and DP-treated groups. (TIF) [file pone.0125983.s003.tif]

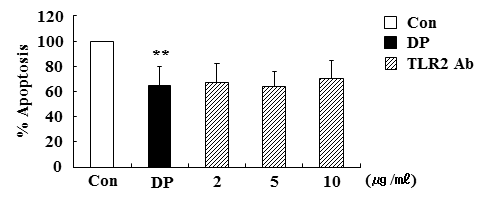

Supplement: S4 Fig — Normal neutrophils (n = 3) were pretreated in the absence and presence of anti-TLR2 blocking antibodies in the indicated concentration for 1 h, after which the cells were incubated for 24 h in the absence and presence of DP (10 μg/ml). Apoptosis was analyzed by measuring the binding of annexin V-FITC and PI. Data are expressed as the means ± SD and are presented relative to the control, which was set at 100%. **p < 0.01 indicates a significant difference between the control and DP-treated groups. (TIF) [file pone.0125983.s004.tif]

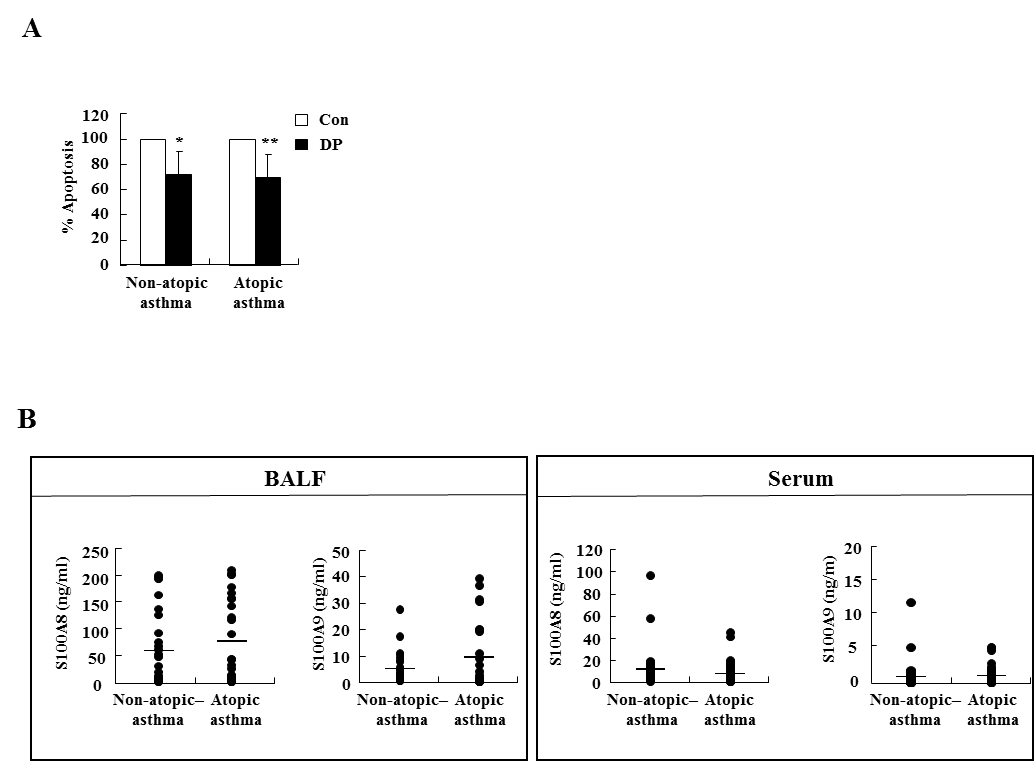

Supplement: S5 Fig — (A-B) Data are presented by classifying the results from Fig 5A (A) and from Fig 6A (B), depending on non-atopic and atopic asthma. (TIF) [file pone.0125983.s005.tif]

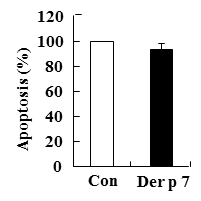

Supplement: S6 Fig — Normal neutrophils (n = 7) were incubated for 24 h with and without Der p 7 (10 μg/ml). Apoptosis was analyzed by measuring the binding of annexin V-FITC and PI. Data are expressed as the means ± SD and are presented relative to the control, which was set at 100%. (TIF) [file pone.0125983.s006.tif]
